# Brain Kynurenine and BH4 Pathways: Relevance to the Pathophysiology and Treatment of Inflammation-Driven Depressive Symptoms

**DOI:** 10.3389/fnins.2018.00499

**Published:** 2018-07-24

**Authors:** Sylvie Vancassel, Lucile Capuron, Nathalie Castanon

**Affiliations:** ^1^UMR 1286, Laboratory of Nutrition and Integrative Neurobiology (NutriNeuro), INRA, Bordeaux, France; ^2^UMR 1286, Laboratory of Nutrition and Integrative Neurobiology (NutriNeuro), Bordeaux University, Bordeaux, France

**Keywords:** inflammation, neuroinflammation, kynurenines, tetrahydrobiopterin (BH4), monoamines, depressive symptoms, antidepressant treatment, anti-inflammatory strategies

## Abstract

The prevalence of depressive disorders is growing worldwide, notably due to stagnation in the development of drugs with greater antidepressant efficacy, the continuous large proportion of patients who do not respond to conventional antidepressants, and the increasing rate of chronic medical conditions associated with an increased vulnerability to depressive comorbidities. Accordingly, better knowledge on the pathophysiology of depression and mechanisms underlying depressive comorbidities in chronic medical conditions appears urgently needed, in order to help in the development of targeted therapeutic strategies. In this review, we present evidence pointing to inflammatory processes as key players in the pathophysiology and treatment of depressive symptoms. In particular, we report preclinical and clinical findings showing that inflammation-driven alterations in specific metabolic pathways, namely kynurenine and tetrahydrobiopterin (BH4) pathways, leads to substantial alterations in the metabolism of serotonin, glutamate and dopamine that are likely to contribute to the development of key depressive symptom dimensions. Accordingly, anti-inflammatory interventions targeting kynurenine and BH4 pathways may be effective as novel treatment or as adjuvants of conventional medications rather directed to monoamines, notably when depressive symptomatology and inflammation are comorbid in treated patients. This notion is discussed in the light of recent findings illustrating the tight interactions between known antidepressant drugs and inflammatory processes, as well as their therapeutic implications. Altogether, this review provides valuable findings for moving toward more adapted and personalized therapeutic strategies to treat inflammation-related depressive symptoms.

## Introduction

Depression currently represents a global public health concern. Not only its prevalence is steadily increasing worldwide but also it relates to a stronger risk of morbidity and death (World Health Organization, 2017). In addition, despite advances made in the treatment of depression, at least one third of depressed patients fail to respond to conventional medications (Rush et al., 2006). Further contributing to the global burden of depression, most of the chronic diseases whose prevalence is also continuously rising, including metabolic, autoimmune and cardiovascular diseases, are associated with an increased risk of depression (Luppino et al., 2010; Capuron et al., 2017; Zuzarte et al., 2018). The latter impairs the quality of life of affected patients and emerges as a potent contributor of subsequent medical complications (Penninx et al., 2013). Thus, a better knowledge of the pathophysiological bases of depression and identification of new targets relevant for therapeutic advances are still clearly needed.

While different neurobiological systems are likely involved in the pathophysiology of depression, this review will summarize evidence that supports a main role for inflammatory processes. First dedicated to decipher the intricate relationship between the innate immune system and the brain, initial research performed in the field of psychoneuroimmunology started to identify the molecular mechanisms underlying the behavioral and neuropsychiatric consequences of inflammation (for review Dantzer et al., 2008; Capuron and Miller, 2011). Meanwhile, converging clinical studies reported a link between increased circulating levels of inflammatory factors and greater risk of developing mood alterations (Capuron et al., 2002; Evans et al., 2005). Altogether, these findings have fostered still on-going investigations aiming to further identify the neurobiological pathways targeted by cytokines and in turn mediating their neuropsychiatric effects. Moreover, in line with recent data suggesting that elevated basal inflammation, as reported in obesity and metabolic syndrome, may contribute to impair the therapeutic efficiency of conventional antidepressant treatment (Kloiber et al., 2007; Toups et al., 2013), a corollary question arises as to whether inflammatory factors, their signaling pathways and/or neurobiological targets, may represent potential targets for new pharmacological interventions. This review will discuss those issues in the light of recent findings providing a deeper mechanistic understanding of the role of inflammation in the pathophysiology of depression and relevant insights for novel therapies.

## The Inflammatory Hypothesis of Depression

Although progressively extended to several mental illnesses, including bipolar disorders, anxiety disorders or schizophrenia (Potvin et al., 2008; Hoge et al., 2009; Barbosa et al., 2013), the notion that inflammation may be involved in the pathophysiology of neuropsychiatric symptoms was particularly studied in the context of depression (Raison et al., 2006; Capuron and Miller, 2011). The first studies strongly supporting this notion report higher circulating levels of inflammatory mediators, including C-reactive protein (CRP), cytokines (particularly interleukin-6, IL-6) and different chemokines, in depressed individuals compared to healthy controls (Dowlati et al., 2010; Leighton et al., 2018). Moreover, longitudinal investigations reveal that higher inflammatory profiles predict the development of depressive symptoms (Valkanova et al., 2013; Smith et al., 2018). To move from correlation to causality, chronic cytokine administration has been shown to induce depression in up to 50% of medically ill patients undergoing cytokine immunotherapy (Musselman et al., 2001; Capuron et al., 2002; Kawase et al., 2016). Similarly, direct administration of cytokines, or cytokine inducers, such as lipopolysaccharide (LPS), to healthy volunteers triggers depressive symptoms (Schedlowski et al., 2014; Engler et al., 2017). Consistent with clinical findings, both acute or chronic immune challenges in rodents induce sustained depressive-like and anxiety-like behaviors (Merali et al., 2003; Frenois et al., 2007; Moreau et al., 2008; Klaus et al., 2016). Conversely, anti-inflammatory compounds reduce these behaviors in animal models of inflammatory diseases (Llorens-Martin et al., 2014; Zaminelli et al., 2014; Norden et al., 2015). Likewise, directly targeting specific inflammatory cytokines decreases mood alterations in both humans and rodents (Tyring et al., 2006; Kekow et al., 2010; Haji et al., 2012; Bayramgürler et al., 2013; Fleming et al., 2015).

Inflammation-induced behavioral changes have been shown to rely on a large communication network allowing inflammatory cytokines [e.g., IL-1β, IL-6, tumor necrosis factor (TNF)-α], which are released peripherally by activated immune cells, to reach the brain through humoral, nervous and chemical pathways and to locally induce production of brain cytokines by activated microglia, the immune cells of the brain. These cytokines in turn influence pathways involved in the regulation of behavior and mood, including neurotransmitter metabolism, neuroendocrine function and neural plasticity (Castanon et al., 2004; Frenois et al., 2007; Dantzer et al., 2008; Capuron and Miller, 2011). These neuroimmune interactions not only coordinate the immune response, but also the development of adaptive behavioral changes collectively referred to as sickness behavior. These changes, which include weakness, listlessness, malaise, anorexia and fatigue, have been shown to be necessary for recovery, by helping the body to actively fight against aggressions. Inflammatory activation is usually transient and controlled by anti-inflammatory mechanisms to warrant time-limitation and reversibility of sickness behavior. Conversely, failure of those control mechanisms that allows development of sustained inflammation has been shown to represent a major leading cause of inflammation-related mood alterations (Dantzer et al., 2008; Capuron and Miller, 2011; Castanon et al., 2014; Capuron and Castanon, 2017). Indeed, if inflammation initially serves a protective function in controlling infection and promoting tissue repair, it can in the long run interfere with brain neurotransmission and cause tissue damages, what ultimately contributes to promote sustained behavioral and mood alterations. Interestingly, compelling clinical studies revealed that patients under cytokine immunotherapy display symptoms spanning multiple dimensions from neurovegetative (e.g., fatigue, decreased tone and motivation) to neuropsychiatric (e.g., depressed mood, anxiety, cognitive alterations) levels (Musselman et al., 2001; Capuron and Miller, 2011; Capuron and Castanon, 2017). Importantly, these symptoms differ in their time-course and responsiveness to prophylactic treatment with classical antidepressant drugs, particularly those targeting serotonin neurotransmission. In particular, neurovegetative symptoms appear early after initiation of cytokine immunotherapy and in a large proportion of patients. On the contrary, neuropsychiatric symptoms progressively develop after several weeks of cytokine administration and only affect 30–50% of patients. These last symptoms can be prevented by antidepressant treatment, in contrast to neurovegetative symptoms that are not, or only minimally, responsive to this intervention (Musselman et al., 2001; Capuron and Miller, 2011). Altogether, these findings suggested the involvement of distinct underlying mechanisms.

Strong support for this assumption came from studies performed in immune-challenged animals in which it is possible to experimentally dissociate sickness behavior, occurring early after the immune stimulation, from protracted depressive-like behaviors (Merali et al., 2003; Frenois et al., 2007; Moreau et al., 2008; Klaus et al., 2016). First attempts to assess the respective underlying neurobiological mechanisms have concentrated on the same preclinical models. In that context, it is worth mentioning that some of the behavioral changes characterizing sickness behavior, particularly motor slowing, could interfere with the measure of depressive-like behaviors when assessed in paradigms based on motor responses. This potential methodological bias has, however, been circumvented by using several reliable and complementary behavioral tests modeling different core symptoms of depression, and by testing mice in these tests only once they have totally recovered from sickness behavior (Frenois et al., 2007; Godbout et al., 2008; O’Connor et al., 2009c). Interestingly, this experimental design in LPS-challenged mice enabled to show a neuroanatomical dissociation between the brain structures that underlie LPS-induced sickness and depressive-like behaviors, respectively (Frenois et al., 2007). It could still be argued that some inconsistencies exist across the literature (see Biesmans et al., 2013) and that a model of acute immune activation with LPS is not necessarily relevant to the clinical situation. Several studies performed in models of chronic inflammation, which can be claimed as more suitable, however, confirmed the presence of sustained depressive-like behaviors while sickness behavior was not anymore detectable nor levels of circulating cytokines elevated (Moreau et al., 2008; O’Connor et al., 2009a,b). Together with clinical studies, all these models turned out to be very useful to progress in the identification of the neurobiological bases of inflammation-related depressive symptoms.

## Neurobiological Bases of Inflammation-Related Depressive Symptoms

Mounting clinical and preclinical findings led us and others to propose that the multidimensional inflammation-related symptoms may rely on the ability of inflammatory cytokines to alter important metabolic pathways, namely kynurenine and tetrahydrobiopterin (BH4) pathways, which in turn can impair neurotransmission of monoamines, particularly serotonin, glutamate and dopamine, involved in mood regulation (Dantzer et al., 2008; Capuron and Miller, 2011; Capuron and Castanon, 2017; Haroon et al., 2017).

### The Kynurenine Pathway: At the Crossroad Between Inflammation and Mood

**FIGURE 1:**
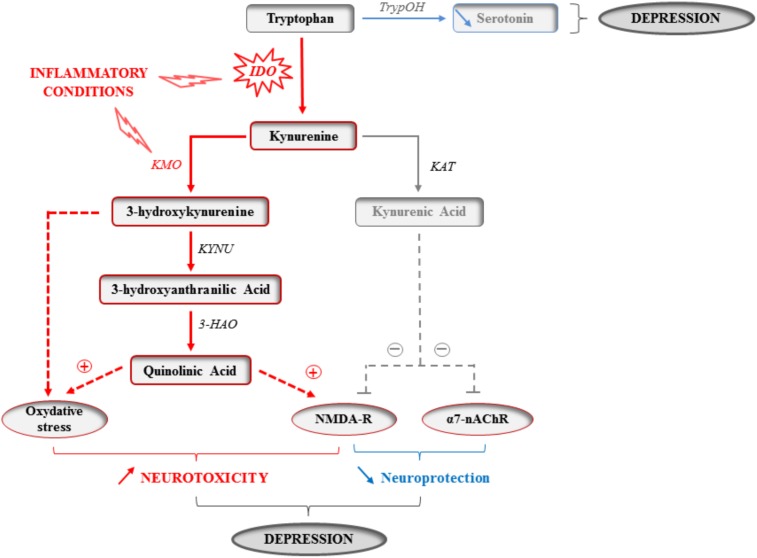
Kynurenine pathway activation in inflammatory conditions. The enzymatic activity of the indoleamine 2,3-dioxygenase (IDO) is increased by inflammatory cytokines in activated monocytes, macrophages and brain microglia in inflammatory conditions. The essential amino acid tryptophan is therefore used for the synthesis of kynurenine, and this at the expense of the synthesis of the monoamine serotonin that directly depends on the availability of its precursor and limiting factor tryptophan. IDO activation might therefore impair serotonin neurotransmission. Kynurenine is then used to produce different neuroactive glutamatergic metabolites, including kynurenic acid, which is neuroprotective by acting on both glutamatergic NMDA and α7-nicotinic acetylcholine (α7-nAChR) receptors, and 3-hydroxykynurenine and quinolinic acid that are rather neurotoxic by promoting oxidative stress and/or activating NMDA receptors. As the activity of the kynurenine monooxygenase (KMO) that synthesizes 3-hydroxykynurenine is increased in activated microglia by inflammatory cytokines, increased production of kynurenine is ultimately associated with skewing of the kynurenine metabolic balance toward increased neurotoxicity. By impairing serotonin neurotransmission and promoting neuronal damages, cytokine-induced kynurenine pathway activation can therefore contribute to the development of inflammation-driven depressive symptoms. TrypOH, tryptophan hydroxylase; KAT, kynurenine aminotransferase; KYNU, kynureninase; 3-HAO, 3-hydroxyanthranilic acid oxygenase.

The metabolism of the essential amino acid tryptophan produces an array of crucial factors able of regulating key physiological processes linked to behavior and mood, particularly serotonin. Tryptophan metabolism along the kynurenine pathway (KP) accounts for most of the tryptophan that is not used for protein synthesis, and ultimately leads to the production of several neuroactive metabolites, including 3-hydroxykynurenine (3-HK) and quinolinic acid (QA), which are able to stimulate *N*-methyl-D-aspartate (NMDA) glutamatergic receptors and promote oxidative stress, and kynurenic acid (KA) that rather displays neuroprotective properties (**Figure 1**) (Schwarcz and Stone, 2017). Since microglia preferentially produce QA, while KA is synthesized by astrocytes, neurotoxicity prevails in conditions of immune activation. Accordingly, increased plasma and/or cerebrospinal fluid QA levels have been reported in a plethora of conditions that encompass inflammatory and neurodegenerative damages, together with an increased prevalence of mood symptoms (Capuron et al., 2011; Bay-Richter et al., 2015; Lovelace et al., 2017; O’Farrell and Harkin, 2017; Schwarcz and Stone, 2017). More importantly, KP activation correlates with both stretch of brain damages and severity of neuropsychiatric symptoms (Capuron et al., 2002; Raison et al., 2010; Savitz et al., 2015a,b). These findings prompted a surge of interest for the involvement of KP enzymes and metabolites in inducing these symptoms, starting by the enzyme indoleamine 2,3-dioxygenase (IDO) that catalyzes the first and rate-limiting step of tryptophan metabolism along the KP (Lestage et al., 2002; Moreau et al., 2005; André et al., 2008). For example, it was shown in mice that inoculation with *Bacillus Calmette-Guerin* (BCG) chronically increases both peripheral and brain IDO activity, this activation paralleling development of sustained depressive-like behaviors (Moreau et al., 2008; O’Connor et al., 2009a,b). Aged mice (Godbout et al., 2008; Wynne et al., 2010), or mice exhibiting constitutive microglial over-activation (Corona et al., 2013) also display sustained cytokine production after an immune challenge, together with protracted brain IDO expression and depressive-like behavior (Godbout et al., 2008; Wynne et al., 2010; Corona et al., 2013). Similarly, a link between inflammation-related brain IDO activation and depressive-like behavior has been reported in other chronic inflammatory conditions, such as murine models of obesity (André et al., 2014; Dinel et al., 2014). Importantly, direct KP activation within the brain is sufficient to alter emotional behaviors in rodents, particularly when it occurs in key brain areas for behavior and mood, particularly the hippocampus (Henry et al., 2009; Fu et al., 2010; Park et al., 2011; Dobos et al., 2012; Lawson et al., 2013). By using mice with either genetic or systemic inhibition of IDO and submitted to an immune challenge, we and others described the causal role of IDO activation in the induction of depressive-like and anxiety-like behaviors (Godbout et al., 2008; O’Connor et al., 2009a,b,c; Salazar et al., 2012; Corona et al., 2013; Gibney et al., 2013; Xie et al., 2014; Castanon, 2015). This induction can be related to the potential for IDO to negatively impact serotonin synthesis by decreasing bioavailability of tryptophan. However, the fact that immune activation is actually associated with increased serotonin turnover (Godbout et al., 2008; O’Connor et al., 2009c; Gibney et al., 2013; Parrott et al., 2016a), regardless of concomitant IDO activation (O’Connor et al., 2009c), weakens this hypothesis. These findings may rather suggest intervention of other cytokine-induced alterations in serotonin neurotransmission, including modulation of serotonin transporter and receptors (Zhu et al., 2006). Follow-up work is therefore needed to deeply study the consequences of KP activation on the temporal and spatial patterns of serotonin-related processes.

An alternative explanation for the involvement of KP activation in inflammation-related mood symptoms is the generation of neurotoxic glutamatergic kynurenine metabolites, as they have been clinically related to the severity of mood alterations (Capuron et al., 2011; Bay-Richter et al., 2015; Haroon et al., 2017; Schwarcz and Stone, 2017). In line with the well-established role of altered neuron integrity and/or function in subtending the clinical outcomes of neurodegenerative diseases, neuronal damages have been also linked to the development of inflammation-induced depressive symptoms (Dantzer and Walker, 2014). This may particularly involve the NMDA receptors, which have recently drawn much attention in the field of depression research (Dantzer and Walker, 2014; Haroon et al., 2017). Supporting further this notion, preclinical studies report that depressive-like behaviors induced by LPS challenge (Parrott et al., 2016a,b) or related to neuropathic pain (Laumet et al., 2017) are associated with skewing of the kynurenine metabolic balance toward production of neurotoxic metabolites, and this in a region-specific manner with the hippocampus being particularly affected. Moreover, systemic administration of kynurenine (O’Connor et al., 2009c; Salazar et al., 2012; Agudelo et al., 2014) or 3-HK (Parrott et al., 2016a,b) dose-dependently impairs emotional behaviors, whereas mice deficient for IDO are protected against NMDA receptor-mediated excitotoxicity (Mazarei et al., 2013). Of note, NMDA receptor blockade abrogates inflammation-induced depressive-like behavior (Walker et al., 2013), along with selective inhibition of downstream KP enzymes, i.e., kynurenine 3-monooxygenase (KMO) or 3-hydroxyanthranilic acid dioxygenase (HAAO) that ultimately synthesize the NMDA receptor agonist QA (Parrott et al., 2016b; Laumet et al., 2017). Interestingly, this especially improves depressive-like behaviors related to clinical behavioral despair (i.e., immobility in the tail suspension test), while being less efficient on behavioral changes rather modeling anhedonia (i.e., sucrose preference) (Parrott et al., 2016b). These results are particularly relevant to help identifying the neurobiological mechanisms that, respectively, underlie the different inflammation-related depressive symptoms. As each behavior/symptom is functionally subtended by preferential activation of selective neuronal pathways in discrete brain regions, such dimensional dissociation may rely on the regional differences of KP activation reported in those experimental studies (Parrott et al., 2016a,b; Laumet et al., 2017). While LPS-induced brain expression of cytokines is largely region-independent, the 3-HK/KA ratio is significantly increased in the hippocampus, but not central amygdala or nucleus accumbens (Parrott et al., 2016a). Consistent with these preclinical findings, post-mortem studies of depressed patients link severe depression with increased microglial QA detection in selective cortical subregions (Steiner et al., 2011). Conversely, a higher neuroprotective index, as reflected by elevated KA/QA ratio, positively correlates with hippocampal volume in clinically depressed but non-medicated patients (Savitz et al., 2015a). Otherwise, the above-mentioned dissociation between KP activation and development of specific depressive symptoms may rather reflect involvement of other metabolic pathways and/or neurotransmission systems (Capuron and Castanon, 2017). This assumption is nicely supported by a recent study showing that inflammation-induced impairment of motivation-driven behaviors is independent from IDO activation since it persists in LPS-treated IDO deficient mice (Vichaya et al., 2018). With regard to inflammation-related motivational deficits, the BH4 pathway that can ultimately impair dopamine neurotransmission appears as a likely candidate (Felger and Treadway, 2017). This is supported by data obtained in mice deficient in BH4, which exhibit dopamine-related behavioral alterations (Choi and Tarazi, 2010). Both alternatives are not necessarily disconnected since KA has been shown to modulate striatal dopaminergic tone, by acting on α7-nicotinic acetylcholine receptors (Wu et al., 2007), while pharmacological activation of these receptors mitigates anhedonia in a mouse model of chronic stress (Zhao et al., 2017). Moreover, clinical data report an inverse association between circulating KA/QA ratio and the degree of anhedonia in depressed patients (Savitz et al., 2015a).

### The BH4 Pathway: A Key Player in Neurobiological Modulation of Depressive Symptoms

**FIGURE 2:**
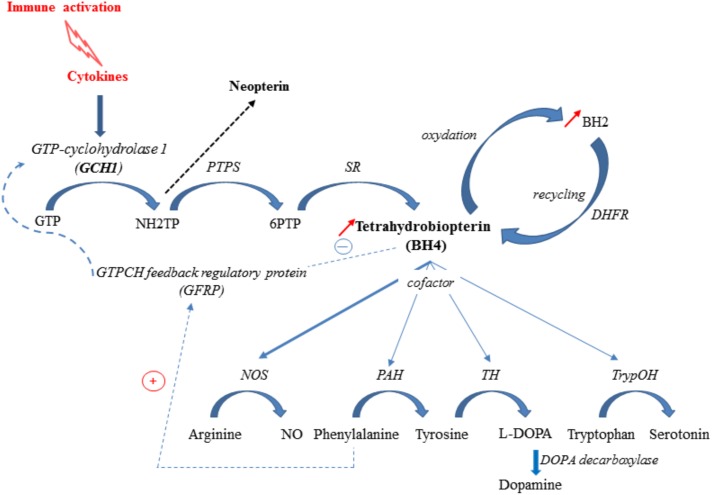
Inflammation-associated BH4 pathway. Cytokine production elicited in response to an immune challenge induces GTP-cyclohydrolase-1 (GCH1), 6-pyruvoyl-tetrahydropterin synthase (PTPS) and sepiapterin reductase (SR) activation (*de novo* synthesis pathway), leading to tetrahydrobiopterin (BH4) synthesis. GCH1 activity is modulated by the interaction of GTP-cyclohydrolase feedback protein (GFRP) and effectors molecules, BH4 and phenylalanine. BH4 is rapidly oxidized to BH2, which is subsequently reduced back to BH4 by the enzyme DHFR, representing a salvage pathway. In Human, PTPS becomes rate-limiting under inflammatory challenge and drives neopterin formation to the detriment of BH4. Under inflammatory conditions, BH4 is preferentially used as cofactor for NOS (nitric oxide synthase) activity, for synthesis of nitric oxide (NO), leading to a lower availability for PAH (phenylalanine hydroxylase), TH (tyrosine hydroxylase) and TrypOH (tryptophan hydroxylase) activities and reduced synthesis of monoamines. NH_2_TP: 7,8Dihydroneopterin triphosphate; 6PTP: 6Pyruvoyl-tetrahydrobiopterin.

BH4 is a pivotal cofactor for the optimal functioning of the nitric oxide synthase isoforms (NOS) and three aromatic amino acid hydroxylases: phenylalanine hydroxylase (PAH), tryptophan hydroxylase (trypOH) and tyrosine hydroxylase (TH) (Thöny et al., 2000; Sumi-Ichinose et al., 2001). Accordingly, it is required for the synthesis of dopamine, serotonin and nitric oxide from the essential amino acids tyrosine, tryptophan and arginine, respectively (Sumi-Ichinose et al., 2001). BH4 *de novo* synthesis involves the sequential activation of three enzymes, the first one, GTP-cyclohydrolase 1 (GCH1) being the rate-limiting step (Thöny et al., 2000) (**Figure 2**). The activity of this enzyme, which is co-localized with the monoamine-containing cells, has been shown to differ according to the brain area (Sawada et al., 1986; Nagatsu et al., 1995; Dassesse et al., 1997). Inflammatory cytokines, including IL-1β, interferon-γ and TNF-α, are able to induce both GCH1 expression and activity, therefore increasing BH4 synthesis (Shi et al., 2004). At the post-translational level, GCH1 is inhibited by BH4 and stimulated by phenylalanine, through its complex formation with the cyclohydrolase feedback regulatory protein (GFRP) (Neurauter et al., 2008). This process ensures tightly keeping BH4 levels within a physiological range in the body. BH4 is also oxidized to BH2, which can, however, be conversely converted back to BH4 by the dihydrofolate reductase (DHFR) (Harada et al., 1993). *In fine*, the net cellular bioavailability of BH4 likely results therefore from the balance between its *de novo* synthesis, its oxidation to BH2, and its regeneration by DHFR.

Several clinical conditions have been associated with a defect in the BH4-pathway, mainly linked to alterations affecting the different enzymatic steps. Mutation of GCH1 results in greatly reduced BH4 level, which has been shown to cause neurological diseases, such as dopamine-responsive dystonia (Ichinose et al., 1994; Müller et al., 2002). Mice genetically deficient for BH4 (*hph1* model) display decreased dopamine levels and increased depressive-like behaviors (Nasser et al., 2014). Supporting preclinical findings, patients with GCH1 deficiency have been shown to exhibit an increased frequency of psychiatric disorders, including depression and anxiety (Van Hove et al., 2006; Trender-Gerhard et al., 2009). Moreover, we have recently reported that variations in markers of GCH1 activity correlate with neurovegetative symptoms and inflammatory factors in elderly persons (Capuron et al., 2011). Low BH4 levels have also been found in postmortem brains of subjects with a history of severe depression (Blair et al., 1984). Conversely, raised urine and plasma total biopterin levels are measured in depressed patients (Duch et al., 1984; Garbutt et al., 1985; Knapp and Irwin, 1989; Hashimoto et al., 1994; Abou-Saleh et al., 1995), suggesting impaired BH4 metabolism. In addition, reduced GCH1 activity, assessed through the increased phenylalanine/tyrosine ratio, has been reported in depressive patients responding to electroconvulsive therapy (Anderson et al., 1994). This phenylalanine/tyrosine ratio is also used as indicator of BH4 availability and PAH activity and may serve therefore as an indirect biomarker of dopamine and norepinephrine synthesis (Neurauter et al., 2008; Capuron et al., 2011).

In Human, low PTPS activity directs the production of neopterin at the expense of BH4 in conditions of GCH1 stimulation, such as inflammation. Neopterin is therefore considered as a marker of cell-mediated immunity in inflammatory conditions (Murr et al., 2002). Increased phenylalanine levels and phenylalanine/tyrosine ratio is reported in patients suffering from chronic inflammatory conditions, this increase being correlated with neopterin concentrations (Neurauter et al., 2008; Ploder et al., 2008; Zangerle et al., 2010; Murr et al., 2014; Hirayama et al., 2016; Ormstad et al., 2016). It is well known that inflammatory stimulation activates the inducible NOS, which considerably increases the use of BH4 for optimal enzymatic activity, and induces formation of large amounts of oxygen radicals that in turn contribute to the oxidative loss of BH4 (Werner et al., 2003). Both increased use and loss of BH4 driven by a chronic inflammatory state may synergistically act to alter the function of BH4-dependent enzymes and then compromise the biosynthesis of monoamines, which may contribute to development of mood disorders (Neurauter et al., 2008; Felger et al., 2013a,b).

Preclinical studies show changes in brain dopamine and serotonin, and/or respective metabolite levels after an immune stimulation (Kamata et al., 2000; Kumai et al., 2000; Kitagami et al., 2003; Sato et al., 2006). Interestingly, inflammation-induced impairment of dopamine neurotransmission, which may involve induction of oxidative damages, has been proposed as a potential mechanism underlying motivational changes reported in LPS-challenged mice (Vichaya et al., 2018). Clinical studies also reveal some functional alterations of the dopaminergic reward system, in association with impairment in motivation and motor slowing (Capuron et al., 2007; Eisenberger et al., 2010; Capuron et al., 2012). Similar blunting of neural responses to reward has been observed in condition of dietary depletion of amino acid precursors for dopamine synthesis (Bjork et al., 2014). Decreased dopamine synthesis and release have been confirmed using microdialysis in monkeys and mice under inflammatory conditions (Felger and Treadway, 2017 for review). Some evidence also shows that inflammatory cytokines may alter the presynaptic dopamine storage through changes in expression and function of the vesicular monoamine transporter (VMAT2) (Kazumori et al., 2004). Moreover, functional changes of the dopamine reuptake pump, DAT, have been suggested in neuroinflammatory conditions associated to HIV infection or prenatal LPS challenge (Gelman et al., 2006; Tien et al., 2013). Then, a reduced presynaptic vesicular storage and/or altered DAT-reuptake of dopamine could together lead to reduced dopamine turnover in inflammatory conditions.

Based on these data, the BH4 pathway is emerging as an important regulator for a number of symptoms and pathologies associated with over-production of inflammatory mediators. Moreover, as previously mentioned recent data suggest a link between KP and BH4 pathways that could act synergistically upon inflammatory conditions to compromise monoamine synthesis. Thus, increased xanthurenic acid, a metabolite of 3-HK, has been shown to directly lower BH4 biosynthesis by inhibiting sepiapterin reductase (Haruki et al., 2016). Similarly, it has been suggested that a concurrent upregulation of kynurenines and BH2 production may lead to a combined up-regulated activity of NOS (by kynurenines) and decreased availability of BH4, as NOS cofactor (Oxenkrug, 2011). Such a combination results in an uncoupling of NOS and consequently reduced production of NO in favor of “inflammogenic” reactive oxygen species (ROS), which cause further oxidative BH4 loss. Altogether, these data strongly support the role of both kynurenine and BH4 pathways in development of inflammatory-related depressive symptoms.

## Therapeutic Implications

One of the major issues when dealing with depressive disorders is the heightened resistance to standard antidepressant therapy (Rush et al., 2006). In that context, the possibility of alternatively and/or concomitantly targeting inflammatory processes to improve clinical outcomes has recently received particular attention (Schmidt et al., 2016; Colpo et al., 2018; Kappelmann et al., 2018; Shariq et al., 2018; Zuzarte et al., 2018). It has been postulated that most classical antidepressants known to primary act on monoamine neurotransmission [i.e., selective serotonin reuptake blockers: SSRIs, tricyclic antidepressants (TCA) acting on norepinephrine reuptake, and antidepressants acting on both serotonin and norepinephrine reuptake: SNRI] may also act on inflammation since they display anti-inflammatory properties both peripherally and within the brain (Lanquillon et al., 2000; Tynan et al., 2012; Strawbridge et al., 2015; Wiêdłocha et al., 2018). Importantly, they not only reduce circulating cytokine levels, but also downstream activation of the KP (Ara and Bano, 2012; Zoga et al., 2014; Reus et al., 2015), ultimately correcting the imbalance between neuroprotective and neurotoxic kynurenine metabolites (Kocki et al., 2012; Eskelund et al., 2017). Hence, sustained SSRI treatment in rodent models of depression reduces QA levels in different brain regions known to be involved in mood regulation (Eskelund et al., 2017). Moreover, variations in the genes coding for IDO and GCH1 have been shown to predict SSRI treatment outcome in depressive patients (Cutler et al., 2012; Kishi et al., 2012), further supporting a role for both pathways in the therapeutic response. Of note, the immune-modulatory impact of antidepressants differs depending on their class, with SSRI and SNRI drugs being mostly anti-inflammatory, while TCAs rather displaying in some studies pro-inflammatory properties (Hamer et al., 2011; Vogelzangs et al., 2012; Nazimek et al., 2016; Chen et al., 2018). Further highlighting the complexity of the relationship between antidepressants and inflammation, a given antidepressant drug can display both pro- or anti-inflammatory properties depending on what immune parameter is assessed (e.g., cytokine release or activation of specific intracellular signaling pathways) (Horowitz et al., 2014). Mounting evidence suggests that treatment resistance might be predicted by elevated inflammation, whether linked to specific gene variants (Baune et al., 2010), activation of selective intracellular pathways (Horowitz et al., 2014) or the presence of chronic inflammatory conditions/diseases (Luppino et al., 2010; Vogelzangs et al., 2012; Hughes and Kumari, 2017). In support of this notion, conditions such as overweight or obesity, which are characterized by a chronic low-grade inflammatory state together with a higher prevalence of depression, have been recently shown to relate to a greater risk of non-response to conventional antidepressants (Kloiber et al., 2007; Toups et al., 2013; Woo et al., 2016; Jantaratnotai et al., 2017). Interestingly, in those conditions, systemic inflammation is associated with both KP activation, as revealed by increased circulating kynurenine levels and expression of the KP enzymes – notably neurotoxic metabolites – in the adipose tissue of obese subjects (Favennec et al., 2015; Alemán et al., 2017), and mood symptoms (Capuron et al., 2008; Daly, 2013). Similar results were obtained in preclinical models of obesity (Dinel et al., 2011, 2014; André et al., 2014; Boitard et al., 2014; Castanon et al., 2015; Almeida-Suhett et al., 2017; de Cossio et al., 2017). Interestingly, weight loss that reduces inflammation and KP activation (Cancello and Clement, 2006; Alemán et al., 2017) correlates with significant mood improvement (mery et al., 2007; Capuron et al., 2011). Beside KP activation, studies reporting increased neopterin levels in obese (Brandacher et al., 2006; Oxenkrug et al., 2011; Mangge et al., 2014) also suggest the potential involvement of BH4 pathway in obesity-related depressive comorbidity, consistent with impairment of dopamine neurotransmission reported in obesity (Sharma and Fulton, 2013; Krishna et al., 2015).

Compelling evidence supports the notion that anti-inflammatory interventions may be effective as novel antidepressants or adjuvants of conventional antidepressants, as long as inflammation and depressive symptoms are comorbid in treated patients (Strawbridge et al., 2015; Kappelmann et al., 2018; Köhler et al., 2016; Schmidt et al., 2016; Jantaratnotai et al., 2017; Jha and Trivedi, 2018) (**Table 1**). For example, non-steroidal anti-inflammatory drugs (NSAIDs) improve antidepressant treatment outcomes in patients with depressive disorders (Müller et al., 2006; Akhondzadeh et al., 2009). Similarly, NSAIDs administration decreases severity of emotional alterations in several animal models of inflammatory diseases, such as cancer (Norden et al., 2015), Alzheimer’s disease (Llorens-Martin et al., 2014), and Parkinson’s disease (Zaminelli et al., 2014). Significant antidepressant effects of the tetracycline antibiotic minocycline have also been reported in depressed patients compared to placebo (for review Rosenblat and McIntyre, 2018). It is also worth mentioning that natural anti-inflammatory agents, such as ω-3 polyunsaturated fatty acids (PUFAs), have also shown promising results on mood, particularly as add-on therapy with conventional antidepressants (for review Layé et al., 2018). Supporting the link between obesity and depression, this strategy revealed to be particularly effective in depressed patients with low-grade basal inflammation (Rapaport et al., 2016), which is linked to elevated body mass index (BMI). Interestingly, supplementation with ω-3 PUFAs in obese subjects also accentuates weight loss induced by low-calorie diet (Kunesová et al., 2006), weight loss in the context of obesity being associated – as mentioned earlier – with reduced inflammation (Lasselin et al., 2014) and improved depressive symptoms (Emery et al., 2007; Capuron et al., 2011). Current knowledge on the implication of specific inflammatory pathways in driving neuropsychiatric symptoms also offers the potential for targeted anti-inflammatory interventions. Thus, monoclonal antibodies against IL-6, IL-17, and TNF-α display antidepressant effects in chronically inflamed patients with significant depressive symptoms, as well as depressed patients with basal low-grade inflammation (Traki et al., 2014; Gossec et al., 2015; Griffiths et al., 2017; Jha and Trivedi, 2018; Kappelmann et al., 2018). Abundant literature also reports antidepressant effects of anti-TNF-α drugs, such as etanercept or infliximab, in clinical trials (Kekow et al., 2010; Arisoy et al., 2013; Raison et al., 2013; Fleming et al., 2015) and murine models of inflammatory diseases (Haji et al., 2012; Bayramgürler et al., 2013). Of note, targeting TNF-α in treatment-resistant depressed patients was found to improve depressive symptomatology particularly in those with higher baseline inflammation (Raison et al., 2013), which tended to be those with higher BMI. In addition, chronic stress-induced depressive-like behaviors are reduced by TNF-α antagonism through decreased IDO activation (Fu et al., 2016).

**Table 1:** Studies investigating antidepressant interventions targeting inflammation, kynurenine or BH4 pathways.

	Population/model	Treatment	Main outcomes
**Clinical trials**		
Akhondzadeh et al., 2009	*n* = 40 Depressed patients	COX-2 inhibitor (celecoxib)	Improvement of antidepressant treatment
Müller et al., 2006	*n* = 40 Depressed patients	COX-2 inhibitor (celecoxib)	Improvement of antidepressant treatment
Kappelmann et al., 2018 (review and meta-analysis)	Chronic inflammatory conditions	Anti-cytokines	↙Depressive symptoms
Arisoy et al., 2013	*n* = 9 patients Ankylosing spondylitis	TNFα blockers (infliximab)	↙Depression and anxiety scores
Fleming et al., 2015 (databases review*)*	*n* = 464 patients Psoriasis	TNFα blockers	↙Depressive symptoms
Raison et al., 2013	*n* = 60 Depressed patients	TNFα blockers (infliximab)	↙Depressive symptoms
Emery et al., 2007	*n* = 13 Obese women	Weight loss (gastric bypass)	↙Depressive symptoms
			↙Inflammatory blood markers (CRP levels)
Pan et al., 2011	Case report (treatment refractory suicidal ideation)	Sapropterin	Mood improvement
Curtius et al., 1983	Case report	BH4	Mood improvement
Woggon et al., 1984	Case report	BH4	No mood change
**Preclinical studies**		
Norden et al., 2015	Mice (Cancer-related fatigue)	Ibuprofen	↙Depressive-like behavior and fatigue-like (FST/wheel running)
			↙IL-1b and IL-6 in hippocampus
Zaminelli et al., 2014	Rats (rotenone Parkinson model)	Ibuprofen	↙Depressive-like behavior (FST)
Fu et al., 2016	Stressed rats (UCMS)	TNFα blockers (infliximab)	↙Depressive-like behavior
			↙Brain IDO and HAAO mRNA expression
Bayramgürler et al., 2013	Rats	TNFα blockers (etanercept)	↙Depressive-like behavior (FST)
Laumet et al., 2017	Mice (spared nerve injury)	(IL-1RA KMO inhibitor (Ro 61-8048)	↙Depressive-like behavior (FST)
			↙Brain KMO mRNA expression
			↙Depressive-like behavior (FST)
Dobos et al., 2012	Mice (LPS)	1-MT	↙Depressive-like behavior
Corona et al., 2013	Mice (LPS)	1-MT	↙Depressive-like behavior (TST)
Xie et al., 2014	Rat (pilocarpine)	1-MT	↙Depressive-like behavior (FST)
			↙Brain IDO mRNA expression and activity
O’Connor et al., 2009c	Mice	1-MT	↙Depressive-like behavior
Gibney et al., 2014	(LPS)	Minocycline	(TST/FST)
	Rats (restraint stress)	Allopurinol	Brain IDO mRNA expression and activity
			↙Depressive-like behavior (FST)
			↙Circulating kyn/tryp


*COX-2, cyclo-oxygenase-2; UCMS, unpredictable chronic mild stress; FST, forced swim test; TST, tail suspension test; IL-1RA, interleukin-1 receptor antagonist; KMO, kynurenine 3-hydroxylase; 1-MT, 1-methyl tryptophan (IDO inhibitor); LPS, lipopolysaccharide; IDO, indolamine 2,3-dioxygenase; HAAO, hydroxyanthranilic acid oxygenase.*

The possibility of acting on brain neurobiological targets of inflammation, such as the KP, rather than on inflammation itself has also drawn much attention, particularly since global anti-inflammatory strategies are often accompanied by important side effects. Thus, compelling preclinical studies report that reducing KP activation promotes antidepressant effects (Reus et al., 2015; Remus and Dantzer, 2016; Jeon and Kim, 2017; Lovelace et al., 2017; O’Farrell and Harkin, 2017). First promising on-trial strategies include blockade of KP enzymes, particularly IDO and KMO, which has been shown to display antidepressant-like properties whose efficiency varies, however, depending on what symptom domains were assessed (Toledo-Sherman et al., 2015; Laumet et al., 2017; Lovelace et al., 2017; Tashiro et al., 2017). Instead of directly targeting KP enzymes, other studies have rather investigated the possibility of reducing the peripheral kynurenine transport across the blood–brain barrier, as the majority of brain kynurenine comes from the periphery. This procedure has been indeed shown to prevent production of QA by activated microglia (Carrillo-Mora et al., 2010), as well as LPS-induced depressive-like behavior (Remus and Dantzer, 2016). Alternatively, it may be worthwhile to increase synthesis/availability of KA, the neuroprotective kynurenine metabolite (Vécsei et al., 2013). By counteracting the neurotoxic effect of QA and other NMDA receptor agonists, increasing KA formation has been shown to reduce neuronal damages and associated experimentally-induced seizures (Russi et al., 1992; Silva-Adaya et al., 2011). Similar beneficial effects still need to be confirmed regarding inflammation-related depressive symptoms, but these data are already very encouraging. Lastly, in agreement with the literature that links glutamate with mood disorders and highlights antidepressant effects of NMDA receptor antagonists such as ketamine (see for review Haroon et al., 2017), several interesting studies suggest that targeting glutamate activity or preventing QA from activating NMDA receptors also offer additional therapeutic opportunities (Dantzer and Walker, 2014; Reus et al., 2015). Blocking activation of those receptors, which was already shown to protect against chronic stress-induced depressive-like behavior (Li et al., 2011), are similarly effective regarding LPS-induced behavioral alterations (Walker et al., 2013). Moreover, IDO deficient mice are less sensitive to QA-induced neuronal damages (Mazarei et al., 2013). Further supporting the role of NMDA receptor activation as potential therapeutic targets to improve inflammation-related depressive symptoms, antidepressant treatment was recently shown to reduce both stress-induced activation of the KP and changes of NMDA receptor expression (Martín-Hernández et al., 2018). Similarly, reducing NMDA receptor activation by enhancing brain KYNA levels has been proposed as a promising way of counteracting amyloid beta-related neurodegeneration (Carrillo-Mora et al., 2010).

Even if the potential antidepressant effect of targeting the BH4 pathway, for example with oral BH4 supplementation, has been only poorly studied so far and with inconsistent results (Curtius et al., 1983; Woggon et al., 1984), supplementation with synthetic BH4 is already used to treat patients suffering from phenylketonuria (Blau, 2013). Moreover, an interesting case report shows that administrating a BH4 replacement protein improves depressive symptoms in a patient suffering from major depression (Pan et al., 2011). Preclinical data also highlight the possibility of increasing brain BH4 levels through its peripheral administration (Ohashi et al., 2016), which consequently changes serotonin and dopamine metabolism (Brand et al., 1996), TH protein content (Homma et al., 2013), dopamine levels and neuronal activities in the brain (Koshimura et al., 2000). More studies are necessary to test if such BH4-induced changes in dopamine and norepinephrine neurotransmission might underlie the expected behavioral improvement, particularly regarding depression-related anhedonic symptoms.

**FIGURE 3:**
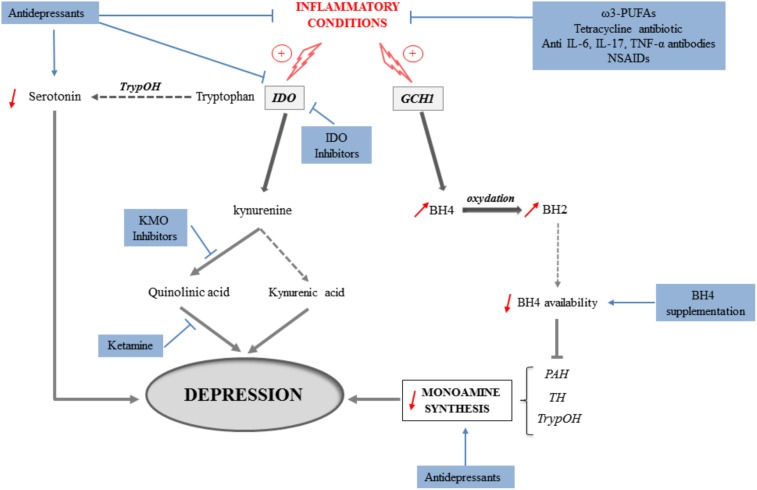
Mechanisms involved in the induction of depressive symptoms in inflammatory conditions (gray arrows) and potential therapeutic implications following pharmacological or nutritional interventions (blue arrows). Activation of the indoleamine 2,3-dioxygenase (IDO) in inflammatory conditions leads to the production of kynurenine from tryptophan, at the expense of serotonin production. The synthesis of serotonin and other monoamines is also impaired because of reduced bioavailability of tetrahydrobiopterin (BH4) that results from the induction of GTP-cyclohydrolase-1 (GCH1) by cytokines. By concomitantly activating the kynurenine monooxygenase (KMO), inflammation promotes the production of the neurotoxic metabolite quinolinic acid, while synthesis of kynurenic acid, rather neuroprotective, is reduced. Both inflammation-induced reduction of monoamine production and increase of neuronal damages ultimately contribute to the development of depressive symptoms. In that context, different therapeutic strategies targeting these mechanisms can be identified. They include: classical antidepressants that aim to increase monoamine synthesis, but also able of reducing inflammation or kynurenine pathway activation; anti-inflammatory interventions with antibiotics, ω3-PUFAs, anti-cytokine antibodies or NSAIDs; IDO or KMO inhibitors; antagonists of the NMDA receptors such as ketamine, and BH4 supplementation. PAH, phenylalanine hydroxylase; TH, tyrosine hydroxylase; TrypOH, tryptophan hydroxylase.

Altogether, these findings clearly show that targeting, either directly or indirectly, inflammation and/or neurobiological mediators shown to underlie inflammation-driven depressive symptoms represents promising new therapeutic strategies (**Figure 3**). These strategies may not only rely on pharmacological approaches using currently available drugs prescribed for their antidepressant properties, but also on the use of still to discover/validate drugs. Regarding the KP, the potential beneficial effects of KP enzyme inhibitors are currently considered in other medical fields, particularly in oncology (see for review Prendergast et al., 2018). Studies carried out in the context of inflammation-driven depressive symptoms should likely benefit therefore from findings reported in other fields. Beside pharmacological approaches, non-pharmacological therapeutic strategies, such as nutritional interventions, may represent a promising alternative associated with reduced risk of complications and economic cost. This particularly includes diet supplementation with natural agents such as ω-3 PUFAs, amino acids or antioxidants, namely compounds known to ultimately protect against inflammation, oxidative stress and/or other neurotoxic insults. Importantly, the opportunity to combine these different therapeutic strategies or rather favor one of them according to the type of depressive symptoms to treat, should broaden the spectrum of action of currently used antidepressant interventions and therefore help improving the management and/or treatment of depressive symptoms associated with inflammation.

## Conclusion

Given the steadily rising prevalence of depression and inflammation-based chronic diseases, their combined negative impact on the etiology of other severe diseases, and the elevated resistance to conventional antidepressants, a better understanding of the pathophysiological mechanisms of inflammation-driven depressive symptoms is urgently required for the identification of new and efficacious therapeutic strategies. Altogether, findings discussed in the present review show that strategies directed to inflammatory processes might be particularly promising, because of their pivotal role in the pathophysiology of depression and their substantial impact on the metabolism of monoamines involved in the regulation of mood. Despite the important advances made over the last decade, several issues are still at stake, from the identification of the best candidates to be targeted within the inflammatory pathways to the determination of the most suitable protocol of treatment (i.e., anti-inflammatory strategies as treatment or co-treatment with conventional antidepressants). Resolving these issues, which clearly depend on the patient’s individual characteristics, constitutes a crucial challenge for the future, as it should allow personalizing antidepressant prescription, and in turn contributing to the development of a precision medicine in psychiatry.

## Author Contributions

All authors listed have made a substantial, direct and intellectual contribution to the work, and approved it for publication.

## Conflict of Interest Statement

The authors declare that the research was conducted in the absence of any commercial or financial relationships that could be construed as a potential conflict of interest.
